# Optimization of RNA extraction methods from human metabolic tissue samples of the COMET biobank

**DOI:** 10.1038/s41598-021-00355-x

**Published:** 2021-10-25

**Authors:** Agathe Nouvel, Jonas Laget, Flore Duranton, Jérémy Leroy, Caroline Desmetz, Marie-Dominique Servais, Nathalie de Préville, Florence Galtier, David Nocca, Nicolas Builles, Sandra Rebuffat, Anne-Dominique Lajoix

**Affiliations:** 1 Biocommunication in Cardio-Metabolism (BC2M), University of Montpellier, 15 avenue Charles Flahault, 34093 Montpellier Cedex 5, France; 2 RD Néphrologie, 2 rue des Muriers, 34090 Montpellier, France; 3 Servier, 50 rue Carnot, 92284 Suresnes Cedex, France; 4 Clinical Investigation Center 1411, Hôpital St Eloi, INSERM, University Hospital of Montpellier, 80 Avenue Augustin Fliche, 34295 Montpellier Cedex 5, France; 5 Department of Endocrinology, Lapeyronie Hospital, University Hospital of Montpellier, 371 avenue du Doyen Gaston Giraud, 34295 Montpellier Cedex 5, France; 6 Department of Digestive Surgery, University Hospital of Montpellier, 80 Avenue Augustin Fliche, 34295 Montpellier Cedex 5, France; 7 Biological Resources Center, Tissue Bank, University Hospital of Montpellier, 80 Avenue Augustin Fliche, 34295 Montpellier Cedex 5, France

**Keywords:** Biomaterials, Endocrinology, Transcriptomics

## Abstract

Constitution of biobank of human tissues requires careful handling and storage of biological material, to guarantee the quality of samples. Tissue preparation is also critical for further applications such as transcriptomic profiling. In this study, our aim was to evaluate the impact of different disruption techniques (FastPrep-24 instrument, GentleMACS dissociator, and syringe/needle) and homogenizing buffers (RLT versus QIAzol) on RNA purity and quality of metabolic tissues (adipose tissues, liver and skeletal muscle) present in the COMET Biobank. For all homogenization methods used and tissue types, the A260/280 ratios reached values ≥ 1.8, which are in the range of what is found in human tissues and cell lines, while the A260/230 ratios were however ≤ 1.8, with the lowest value obtained with GentleMACS Dissociator. In addition, GentleMACS Dissociator combined with QIAzol reagent gave the highest RIN value and 28S/18S ratio for all tissues tested, except for muscle. Performing RT-qPCR, Ct values for different housekeeping genes can be influenced by extraction methods and RNA quality of samples. In conclusion, we have demonstrated that different disruption techniques and homogenizing buffers impact the purity and some quality markers of RNA, and can also impact quantification of mRNAs by RT-qPCR in human metabolic tissues.

## Introduction

With the development of personalized medicine, biobank facilities have clearly increased over the past decades, to meet to the need of therapeutic innovations in human diseases such as the discovery of new drugs or biomarkers^1^. Biobanks contain large sample diversity (tissues, nucleic acids, cells, biological fluids,…), collected from healthy donors and patients with pathologies of interest. The COMET biobank is a biorepository of metabolic tissues collected in morbidly obese patients with different metabolic status (https://cometbiobank.com) and is transferable to the scientific community for research in the field of type 2 diabetes (T2D) and associated comorbidities. The biobank contains a variety of tissues including blood derivatives, adipose tissue, liver and skeletal muscle.

The constitution of a biocollection requires careful handling and storage of biological material, to guarantee samples quality for further applications. Among these applications, molecular profiling using –omics approaches such as genomics, transcriptomics, proteomics and metabolomics, are widely used to generate large-scale data that can be correlated with patients’ clinical features^1^. Nonetheless, among methods aimed at studying nucleic acids, transcriptomic analyses are the most critical techniques since RNA can be degraded by RNases during tissue processing and nucleic acid extraction. During tissue preparation, ex vivo warm ischemia, which refers to the period after surgical extirpation of the biopsy, can influence RNA integrity and possibly, gene expression analysis. Indeed, using transcriptomic analysis, it was found that gene expression profiling can be influenced in certain tissues by ischemia time and temperature^2, 3^. Concerning the delay between tissue removal and snap-freezing, Song et al. found that RNA integrity was unaltered when exposed to ischemia for 1 h^4^, whereas other studies found that RNA quality was stable up to 180 min^5^. Current biobanking practice suggests freezing samples within 30 min, although no consensus arises from the literature, as some authors found that RNA remained intact up to 16 h after resection^6^, while others found no correlation between the duration of ischemia and RNA quality^7^. Regarding the temperature at which the tissue is sampled, RNA quality appears more preserved when the tissue is maintained on ice (“cold-ischemia”) rather than at room temperature or in saline^8, 9^. Despite this, some authors found no influence of temperature^6^. Moreover, RNA stability can vary between tissue types, pathology, and localization, with some tissue being more sensitive than others, such as the liver^10^.

Sample preparation using disruption methods for nucleic acid extraction can also impact RNA quality. Several methods for tissue homogenization can be used, according to the density of the tissue and its intrinsic content of RNases. While non-mechanical methods (e.g., detergent, enzymatic, osmotic lysis,…) are more suitable for single cell homogenization, tissue disruption often requires mechanical techniques, such as high-pressure homogenizer, tissue grinder, bead beating-based homogenization, or rotor/stator-based homogenization^11^. In this study, we have selected three mechanical methods that have shown a high efficiency to disrupt a wide range of cells/tissues to compare their performances across 4 selected metabolic tissues: subcutaneous (SCAT) and visceral (VAT) adipose tissues, which are soft tissues with high lipid content; liver, which is a highly vascularized and relatively fibrous tissue; and skeletal muscle, which is a highly fibrous and hard tissue.

The bead beating method (FastPrep-24 instrument) uses tiny beads that are agitated and accelerated against the tissue, thus creating shear forces that disrupt tissues and release intracellular components^11^. The rotor/stator-based system (GentleMACS dissociator) is based on a rotating paddle which is combined to a fixed stator with a narrow gap between the stator teeth and the rotor to allow tissue dissociation. Another available method is based on forcing the tissue to pass through a narrow gauge needle using a syringe barrel and plunger, which could be an alternative method in the absence of the aforementioned dedicated equipment.

Moreover, homogenizing reagents can also impact the quality and quantity of RNA recovered. Commonly used buffers often contain guanidine (iso)thiocyanate combined with phenol (QIAzol or TRIzol buffer) or β-mercaptoethanol (Qiagen RLT buffer), to allow both cell lysis and RNase inhibition. In this study, we compared two homogenization buffers. For muscle and liver, we used RLT buffer which contains a high concentration of guanidine isothiocyanate, a chaotropic salt which allows cell lysis and binding of RNA to the silica membrane. β-Mercaptoethanol is added to RLT buffer to increase inhibition of RNases. For muscle, a step of protein digestion by proteinase K is included in the extraction kit. For adipose tissues and liver, we used QIAzol reagent, a lysis buffer composed of phenol and guanidine thiocyanate to permit chaotropic disruption of cells and organic extraction of intracellular components. This reagent is recommended for the lysis of fatty tissues but can be applied to all types of tissues.

In the present study, our aim is to evaluate several homogenization methods using different disruption techniques and homogenizing buffers in order to optimize extraction of total RNA for the different metabolic tissues present in COMET Biobank. The influence of these methods on the purity and quality of RNA and subsequent mRNA quantification of housekeeping genes will also be investigated.

## Results

### Comparison of homogenization methods

Using FastPrep-24 Instrument, the number of cycles was determined visually to allow complete dissociation of the tissue. Two to four cycles of 30–40 s separated by incubation on ice were necessary to dissociate the tissues (two cycles for adipose tissues, four for muscle and liver), regardless of the homogenization buffer used. With GentleMACS Dissociator, a single cycle of a predefined program allowed the complete dissociation of all tissues, independent of the buffer used. Alternatively, a complete dissociation of all tissues tested was achieved after several passages through the needle and syringe, except for skeletal muscle.

### Amount of total RNA

**Figure 1:**
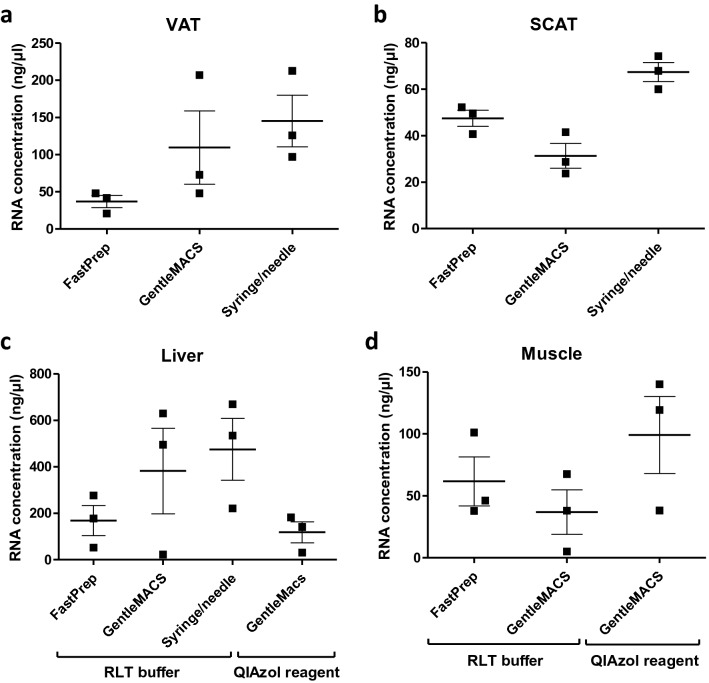
RNA concentration after extraction with different homogenization buffers and methods. RNA concentration was measured using Nanodrop Spectrophotometer at 260 nm. For each condition tested, 20 mg liver and muscle, or 50 mg adipose tissues were subjected to different homogenization buffers and methods. At the end of the extraction protocol, RNA was eluted in 30 µL buffer. Mean values ± SEM are shown for VAT (**a**), SCAT (**b**), liver (**c**), and muscle (**d**). *VAT* visceral adipose tissue, *SCAT* sub-cutaneous adipose tissue. FastPrep for FastPrep-24 Instrument, GentleMACS for GentleMACS Dissociator.

Despite equivalent quantity of tissue sampled in each cryotube, the amount of total RNA extracted (141 ± 27 ng/mL) was highly variable from one tissue biopsy to another (Fig. 1). Homogenization methods influenced RNA levels (*P* = 0.02, Table 1), independently of the tissue type. RNA concentrations reached 79 ± 22 ng/mL for all tissues with FastPrep-24 Instrument and 136 ± 42 ng/mL with GentleMACS Dissociator. Syringe/needle (229.3 ± 74 ng/mL) gave higher RNA levels than FastPrep-24 Instrument (*P* < 0.05) or GentleMACS Dissociator (*P* = 0.06). The type of the tissue also impacted RNA levels (*P* = 0.04, Table 1). Indeed, RNA extraction from liver led to higher amounts of RNA than both adipose tissues (*P* < 0.05) and muscle (*P* = 0.06).

**Table 1:** Estimated least-square means from ANOVA models of RNA quality parameters and RT-qPCR results. The letters indicate significant changes in means (numbers with the same letters are not different). P-value <0.05 are shown in bold. FastPrep for FastPrep Instrument, GentleMACS for GentleMACS Dissociator, Needle for syringe/needle.

Effects and modalities	RNA concentration (ng/mL)		A260/A280 ratio		A260/A230 Ratio		RIN		28S/18S		Ct*	
	Estimated means	P-value	Estimated means	P-value	Estimated means	P-value	Estimated means	P-value	Estimated means	P-value	Estimated mean	P-value
**Disruption**		**0.02**		0.35		**0.001**		0.07		**0.01**		**0.005**
FastPrep	78.8^b^		1.93^a^		1.13^a^		6.3^a^		0.68^b^		24.9^b^	
GentleMACS	120.3^ab^		1.90^a^		0.71^b^		7.7^a^		1.16^a^		25.1^b^	
Needle	252.7^a^		1.97^a^		1.50^a^		6.8^a^		1.26^a^		26.3^a^	
**Tissue**		**0.04**		0.07		0.43		0.16		**0.03**		0.84
Liver	304.5^a^		2.00^a^		1.10^a^		5.9^a^		0.92^b^		25.9^a^	
Muscle	152.1^ab^		1.91^ab^		1.20^a^		7.2^a^		1.55^a^		26.1^a^	
SCAT	48.7^b^		1.87^b^		1.21^a^		7.3^a^		0.83^b^		25.0^ab^	
VAT	97.2^b^		1.92^ab^		0.95^a^		7.4^a^		0.87^b^		24.8^b^	
**Tissue type × disruption**		0.17		0.69		**0.003**		0.32		**0.0005**		0.55
Adipose tissue, FastPrep	42.2^b^		1.87^a^		1.46^ab^		6.2^a^		0.02^b^		24.5^b^	
Adipose tissue, GentleMACS	70.4^b^		1.86^a^		0.74^bc^		8.3^a^		1.30^a^		24.4^b^	
Adipose tissue, Needle	106.3^b^		1.95^a^		1.05^abc^		7.6^a^		1.23^a^		25.8^ab^	
Non-adipose tissue, FastPrep	115.4^ab^		1.99^a^		0.81^bc^		6.5^a^		1.33^a^		25.3^ab^	
Non-adipose tissue, GentleMACS	167.9^ab^		1.93^a^		0.69^c^		7.1^a^		1.03^a^		25.9^a^	
Non-adipose tissue, Needle	397.6^a^		1.99^a^		1.96^a^		6.1^a^		1.35^a^		26.8^a^	

*The effects Gene, Gene × Tissue Type, Gene × Disruption and Gene × Disruption × Tissue Type were included in the model. Results are available in Supplementary Table 1.

### Purity of RNA

**Figure 2:**
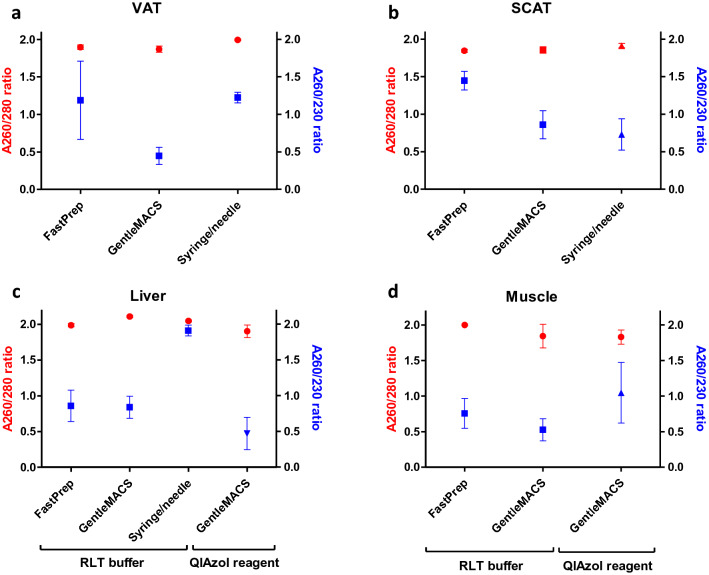
RNA purity after extraction with different homogenization buffers and methods. A260/280 and A260/230 ratios were determined using Nanodrop Spectrophotometer. Mean values ± SEM are shown for VAT (**a**), SCAT (**b**), liver (**c**), and muscle (**d**). *VAT* visceral adipose tissue, *SCAT* sub-cutaneous adipose tissue. FastPrep for FastPrep-24 Instrument, GentleMACS for GentleMACS Dissociator.

According to Nanodrop technical bulletin^12^, a A260/280 ratio of ~ 2.0 and a A260/230 ratio between 1.8–2.2 is expected as indicators of RNA purity. In our experiments, the A260/280 ratio reached a satisfying value of 1.93 ± 0.02 for all samples together (Fig. 2). The ratio was not significantly influenced by disruption techniques (*P* = 0.35). Values were 1.93 ± 0.02 with FastPrep-24 Instrument, 1.91 ± 0.04 with GentleMACS Dissociator and 1.98 ± 0.02 with syringe/needle. There was also differences across tissues (*P* = 0.07). More specifically, the ratio was higher in liver tissue compared to SCAT (*P* < 0.05), which may reflect a difference in RNA nucleotide composition between tissue types^12^.

The A260/230 ratio reached an average of 0.98 ± 0.09, suggesting that few residues of extraction buffer (i.e., phenol and/or guanidine derivatives) remained in the preparation. The A260/230 ratio was more heterogeneous across samples than the A260/280 ratio (Fig. 2). The A260/230 ratio did not vary with tissue type (*P* = 0.43, Table 1); however, it varied according to disruption technique (*P* = 0.001) with a significant interaction with tissue type (adipose vs non adipose tissues) (*P* = 0.003). Indeed, the mean A260/230 ratio was significantly lower with GentleMACS Dissociator (0.71 ± 0.1), than with FastPrep-24 Instrument (1.13 ± 0.17; *P* < 0.05) or syringe/needle (1.33 ± 0.17; *P* < 0.001). In non-adipose tissues, i.e., liver, the syringe/needle gave a higher A260/230 ratio than with GentleMACS Dissociator (*P* < 0.01) or FastPrep-24 Instrument (*P* < 0.05), which was not the case for adipose tissues.

### RNA integrity

**Figure 3:**
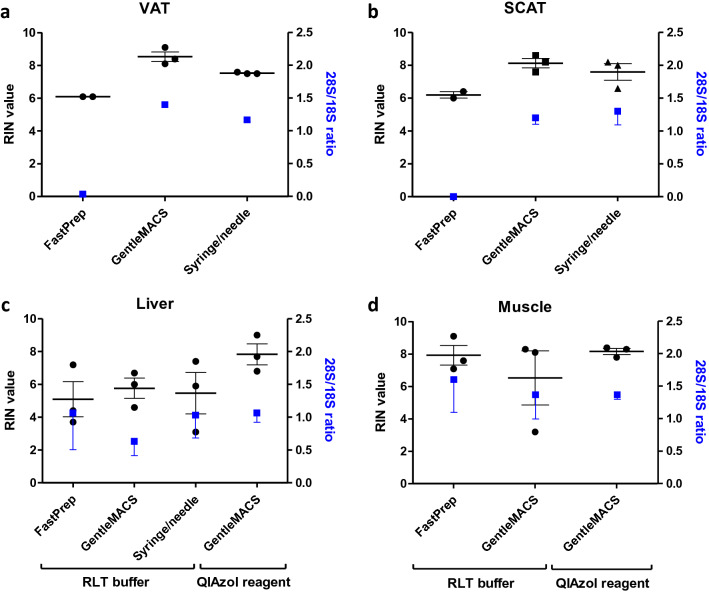
RNA quality obtained with different homogenization buffers and methods. RIN and 28S/18S ratios were determined using Agilent 2100 Bioanalyser and RNA 6000 Nano chips. Mean values ± SEM are shown for VAT (**a**), SCAT (**b**), liver (**c**), and muscle (**d**). *VAT* visceral adipose tissue, *SCAT* sub-cutaneous adipose tissue. FastPrep for FastPrep-24 Instrument, GentleMACS for GentleMACS Dissociator.

In the overall model, RNA Integrity Number (RIN) was not statistically different across disruption methods (*P* = 0.07) or tissues (*P* = 0.16). However, excluding skeletal muscle samples, which were not eligible for dissociation with syringe/needle, we found that RIN was affected by tissue (*P* = 0.02) and disruption method (*P* = 0.014), as higher RINs were observed with GentleMACS Dissociator as compared to FastPrep-24 Instrument (*P* < 0.05). For VAT, RIN achieved 8.5 ± 0.3 with GentleMACS Dissociator versus 6.1 ± 0 with FastPrep-24 Instrument (*P* < 0.01). Concerning SCAT, we observed RIN reaching 8.1 ± 0.3 with GentleMACS Dissociator versus 6.2 ± 0.2 with FastPrep-24 Instrument (*P* < 0.05) (Fig. 3A,B). The syringe/needle method gave intermediate RIN (RIN = 7.5 ± 0.03 for VAT, *P* < 0.01 vs FastPrep-24 Instrument; RIN = 7.6 ± 0.5 for SCAT, NS vs FastPrep-24 Instrument). RIN for liver were lower than the other tissues (*P* < 0.05 vs VAT or SCAT), suggesting that hepatic tissue is more sensitive to RNA degradation than other tissue types (Fig. 3C). Comparing the two homogenization buffers in non-adipose tissues, QIAzol reagent led to higher RIN than RLT buffer (*P* = 0.04). Concerning muscle, homogenization with RLT buffer and FastPrep-24 Instrument gave equivalent RIN as with QIAzol reagent and GentleMACS Dissociator (*P* = 0.5), probably because this tissue is more resistant to degradation during homogenization (Fig. 3D).

**Figure 4:**
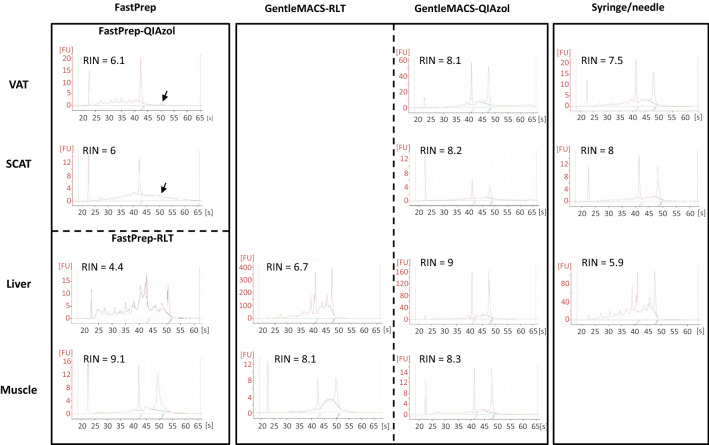
Electropherograms obtained with different homogenization buffers and methods. Electropherograms were obtained using 2100 Bioanalyser and RNA 6000 Nano chips. Typical electrophoretic traces and RIN are shown for VAT, SCAT, liver, and muscle. *VAT* visceral adipose tissue, *SCAT* sub-cutaneous adipose tissue. FastPrep for FastPrep-24 Instrument, GentleMACS for GentleMACS Dissociator.

Concerning the 28S/18S ratio, the overall model showed that the ratio varied with disruption method (*P* = 0.01) and tissue (*P* = 0.03). Again, the 28S/18S ratio was greater with GentleMACS Dissociator or syringe/needle, when compared to FastPrep-24 Instrument (for both methods, *P* < 0.05). In fact, the three techniques provided similar results across tissue types, with the exception of a lower ratio with the FastPrep-24 Instrument in adipose tissues (around 0; *P* < 0.01 vs other conditions) (Fig. 3A,B). Such a difference was due to the absence of 28S RNA band using FastPrep-24 Instrument (Fig. 4). In muscle, the 28S/18S ratio was higher than the three other tissues, *P* < 0.05), confirming that this tissue is less sensitive to degradation (Fig. 3D).

For all tissues, analysis of electropherograms did not highlight the presence of large fragments which could correspond to genomic DNA, regardless the extraction method used (Fig. 4).

### Quantification of mRNA of housekeeping genes

**Figure 5:**
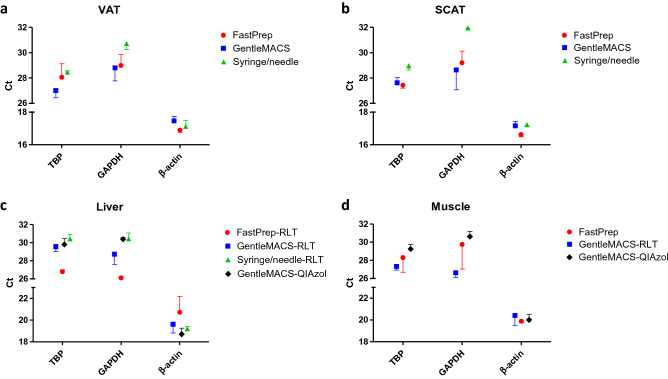
Quantification of mRNA of housekeeping genes according to homogenization buffers and methods used. RT-qPCR was used to amplify three housekeeping genes, TATA-box binding protein (TBP), glyceraldehyde-3 phosphate dehydrogenase (GAPDH), and β-actin. Mean Ct are shown for VAT (**a**), SCAT (**b**), liver (**c**), and muscle (**d**). *VAT* visceral adipose tissue, *SCAT* sub-cutaneous adipose tissue. FastPrep for FastPrep-24 Instrument, GentleMACS for GentleMACS Dissociator.

In all tissues tested, threshold cycles (Cts) were found the lowest for β-actin (*P* < 0.001 vs TBP and GAPDH), followed by TBP (*P* < 0.05 vs GAPDH), and finally GAPDH (Table 1). Comparing tissue types, TBP and GAPDH displayed similar Cts (less than 1Ct difference), while Cts for β-actin were found lower in adipose tissues versus non-adipose tissues (*P* < 0.001) (Fig. 5 and Supplemental Table 1). We observed an effect of disruption methods (*P* = 0.006), with the needle/syringe resulting in higher Cts than other methods. There were higher Cts with QIAzol vs RLT buffer in non-adipose tissues only for GAPDH (*P* = 0.004), when limiting the analysis to the same disruption method (GentleMACS Dissociator).

We also calculated the Ct/RIN ratio as a summary measure including both indications of Ct for a specific transcript and RIN for the sample from which it is derived. This measure allows to better compare Cts obtained from samples with different RIN (Supplemental Table 1). Ct/RIN ratio using GentleMACS Dissociator was significantly lower compared to FastPrep Instrument (*P* < 0.01) and syringe/needle (P < 0.05). The Ct/RIN ratio was also lower with QIAzol (*P* = 0.007) in non-adipose tissues, similarly when limiting the analysis to the GentleMACS instrument (*P* = 0.01). For liver, a significantly lower Ct/RIN ratio could be observed as compared to the three other tissues (*P* < 0.05), confirming that this tissue is more prone to degradation. In addition, an inverse correlation between Cts and RIN could be observed for β-actin in non-adipose tissues (*P* = 0.01), but not for other genes and tissues (*P* > 0.05).

### Impact of RNAlater on RNA quality

**Figure 6:**
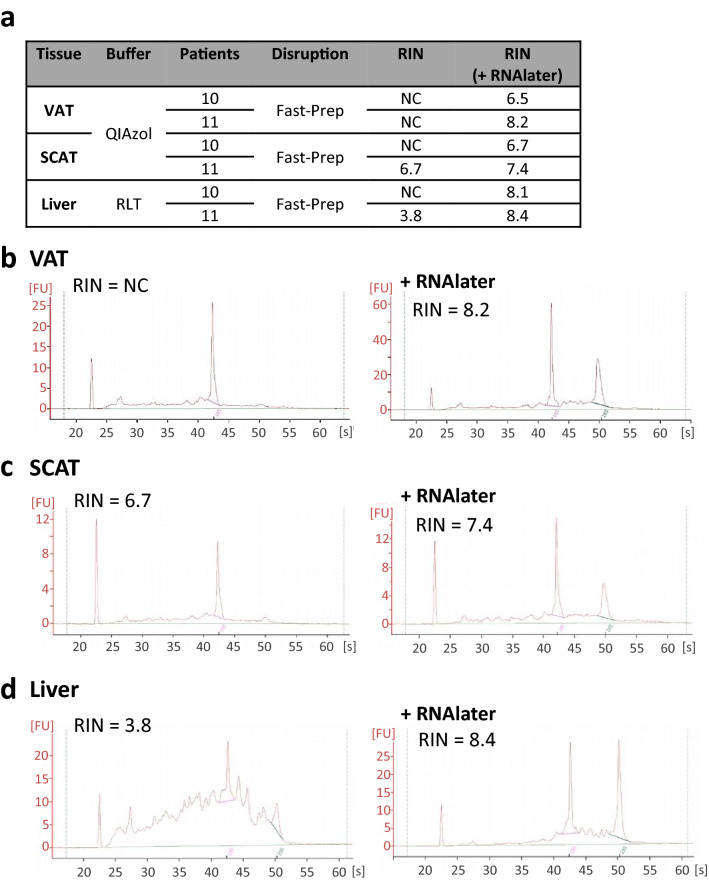
Impact of RNAlater pre-treatment on RNA quality of samples. (**a**) Effect of 24 h-RNAlater pre-treatment on RIN, determined using Agilent 2100 Bioanalyser and RNA 6000 Nano chips. Typical electrophoretic traces and RIN are shown for VAT (**b**), SCAT (**c**), and liver (**d**). VAT, visceral adipose tissue, SCAT, sub-cutaneous adipose tissue. FastPrep for FastPrep-24 Instrument. *NC* non-calculable.

As an alternative to direct freezing of tissues (see methods section), we also evaluated the possibility to immerge the sample in RNAlater, 24 h before freezing. RNAlater, composed of ammonium sulfate and EDTA, is an RNA protector that penetrates the tissue and induces RNases precipitation and inhibition by metal chelation. In addition, tissue stored in RNA later also develop a “hard, rubbery texture, and may be more difficult to homogenize thoroughly than would fresh tissue” (RNAlater product information, Sigma-Aldrich). When using FastPrep-24 Instrument, we observed an improvement of RIN for liver (with RLT buffer) or adipose tissues (with QIAzol reagent), without change in RNA yield (Fig. 6).

### Control of patient effects

Results obtained when controlling the influence of the study design (i.e., samples from the same patient) were virtually identical. Still, because the needle approach was performed on a different set of patients, the conclusions of this method cannot be fully separated from a potential patient effect.

## Discussion

In the present study, we have used human biopsies collected in the frame of COMET biobank to evaluate the impact of several homogenization methods on total RNA recovery. We have found that different disruption techniques and homogenizing buffers influence the purity and some quality markers of RNA and can also impact quantification of mRNAs by RT-qPCR.

Our study shows that homogenization methods clearly impact the recovery of RNAs from human tissues. GentleMACS Dissociator combined to QIAzol reagent appears to be the best method to obtain the highest RIN value and 28S/18S ratio for all the tissues tested, except for muscle. As compared to FastPrep-24 Instrument, GentleMACS Dissociator seems to be more efficient to dissociate human tissues in one cycle, avoiding repeated lysis cycles and thus preserving RNA integrity. Such a difference was also evidenced on skin biopsies (which are very difficult to dissociate), where GentleMACS Dissociator is the only system allowing a complete homogenization of human skin samples, as compared to FastPrep-24 Instrument^13^. Manual disruption by syringe/needle can be a good alternative method which provides RNA with comparable quality to GentleMACS Dissociator, but only in case of soft tissues^14^ or isolated cells^15^. As also shown for skin biopsies^13^, we observe that RNA yields tend to be higher with GentleMACS Dissociator, but no statistical correlation can be found between biopsy weight and RNA levels, i.e., larger biopsies does not necessarily yield to the highest amounts of RNA. This variation can be due to heterogeneity in cellular composition and transcriptional activity in human tissues/organs, with implications on RNA profiles. In human liver biopsies^16^, numerous cell types can be identified by single cell RNA sequencing, including but not limited to hepatocytes, duct cells, endothelial cells, hepatic stellate cells, Kupffer cells, and immune cells. Moreover, the authors also found a transcriptional heterogeneity between hepatocytes according to their spatial localization along the liver lobule^16^. Adipose tissues are also heterogeneous in their composition, as they contain adipocytes, preadipocytes, mesenchymal stem cells, vascular cells, and inflammatory cells^17^. Furthermore, at least three distinct subpopulations of white preadipocytes have been evidenced in mice, with unique gene expression profile^18^. In skeletal muscles, single nuclear sequencing of different nuclei present in synticial myofibers revealed transcriptomic heterogeneity is not related to fiber type differences^19^.

For all homogenization methods used and tissue types, the A260/280 ratio reaches values ≥ 1.8, which are in the range of what is found in human tissues and cell lines^20^, suggesting limited protein contamination. The A260/230 ratio is however ≤ 1.8, with the lowest value obtained with GentleMACS Dissociator. This difference is also observed for DNA extraction, where GentleMACS Dissociator leads to inferior an A260/230 ratio when compared to bead beating-based homogenization methods^21^. A low A260/230 ratio is indicative of the presence of organic compounds such as phenol or guanidine, which is inherent to methods using chaotropic lysis buffers^12^. Nonetheless, guanidine thiocyanate contamination was shown not to interfere with downstream molecular applications, up to 100 mM in RNA samples^22^. Indeed, applications such as cDNA synthesis, in vitro transcription^20^, sequencing^23^, or microarrays^24^ were not affected by low A260/230 ratios.

To assess RNA quality, we used the RIN value, now preferred to the 28S/18S ratio that shows a high variability for RNA extracted from human samples, which is not necessarily related to poor RNA quality^20^. Nonetheless, for RNA extracted from adipose tissues with FastPrep-24 Instrument, the 28S/18S ratio indicated the absence of the 28S rRNA band, preventing the calculation of a RIN value, which may correspond to a mechanical breaking of 28S rRNA. Indeed, this rRNA is more susceptible to be affected by enzymatic degradation, mechanical shearing, or freezing procedure than 18S rRNA, as a result of its important size and the presence of “hidden break” in its polynucleotide chain^25, 26^. In addition, we observed that RINs were lower in liver than in other tissues, probably due to the fact that hepatic tissue contains high levels of RNAses^10^.

RT-qPCR is another method used to evaluate RNA integrity through analysis of mRNA expression of target genes. To avoid underestimation of RNA fragmentation/degradation, we used oligo(dT) primers for reverse transcription, rather than random primers, as previously suggested^27^. In our experiments, we observe a certain degree of variability between patients’ samples, suggesting an impact of their pathophysiological conditions on RNA profile. However, we find some housekeeping genes suitable for normalization of qPCR experiments such as β-actin in adipose tissues and muscle or TBP in liver. Nonetheless, when using human samples, variability between patients can only be minimized, as shown by Kim et al.^28^. The authors observed that no housekeeping gene was found to vary by less than twofold in liver diseases such as cirrhosis or hepatocellular carcinoma.

When normalizing Cts according to RIN (the Ct/RIN ratio), QIAzol and GentleMACS Dissociator gave improved results as compared to other buffer and extraction methods. In addition, an inverse correlation between RIN and Cts is found for β-actin in liver and muscle but not for other genes and tissues. To explain such discrepancies, we suggest that the β-actin mRNA is more sensitive to degradation by RNases or has a shorter half-life in these tissues, as it was shown previously in human cell lines^29^. In addition, we cannot exclude that RNA isolation methods differentially influence the recovery of some mRNA species, as previously suggested in studies comparing phenol extraction or RNA isolation kits^30, 31^. For adipose tissue, Cts remains unchanged regardless RIN observed. This low RIN may not necessarily reflect low quality mRNA despite the observed degradation of 28S rRNA. This suggests that, in adipose tissues, 28S rRNAs undergo degradation by mechanical breaking rather than degradation by enzymatic activity of RNases, with consequential reduction in RIN.

In the literature, the relationship between RIN and subsequent analysis of mRNA expression levels by RT-qPCR is not clear. For instance, a correlation between RIN and apparent expression levels of housekeeping genes is found during the progressive degradation of RNA samples, of unknown tissue origin^26^. Concerning human samples, Imbeaud et al.^20^ found that RNA quality metrics (Degradometer and RIN analysis) are predictive of relative gene expression analysis in RNA samples of disparate quality. In another study performed on a lung biobank^5^, the authors observed that gene expression measurements are more influenced by interpatient differences than other variables. Even for two samples issued from the same individuals, they observed that variability in mRNA expression levels was mostly within the range of a factor of 2.

Nonetheless, Kap et al. propose to divide RNA samples into “fit for purpose” groups according to RIN, with samples having RIN above 5 suitable for RT-qPCR^32^. When RIN are lower than 5, they suggest that samples can be also used for RT-qPCR, but only for amplification of small size amplicons^32^. For genome-wide studies such as microarrays or RNA sequencing, inclusion of samples with highest quality metrics is recommended to avoid experimental bias, as suggested by the literature^32–35^.

We also evaluated an alternative method for sample preparation for biobanking, using a 24 h-incubation in RNA stabilizing reagents like RNAlater before freezing^6^. For COMET biobank, we have chosen direct freezing of tissues to allow several applications to be performed on the same type of sample, such as genomics, proteomics, metabolomics, and histological analysis. However, to avoid tissue damage, we have reduced the time of ex-vivo warm ischemia to a minimum, as the biopsy is immediately collected after removal from the patient. Moreover, we prepared samples at low temperature within a short period of time before snap-freezing (15 or 20 min depending on the tissues). However, using RNA stabilizing reagents have the advantage of avoiding the need of freezing facilities in the clinic surgical block. Nonetheless, the impact of protective reagents is not clear in the literature, as a previous study showed that gene expression analysis is not altered after cold ischemia of 180 min, independently of pre-treatment with RNAlater^5^. Therefore, RNAlater does not necessarily improve RNA quality, if samples are prepared in a limited period of time before freezing. This data is in accordance with our experiments showing good quality of RNA when using QIAzol/ GentleMACS Dissociator. The effect of RNAlater is more obvious in fat and liver tissues when tissue homogenization is performed with FastPrep-24 Instrument, with both tissue types becoming harder and thus more resistant to the stress induced by mechanical dissociation.

In conclusion, we have demonstrated that different disruption techniques and homogenizing buffers impact the purity and some quality markers of RNA isolated from metabolic tissues and can also affect mRNA quantification by RT-qPCR. To optimize quality of RNA preparation, we suggest to prepare human samples at low temperature in a short period of time and to implement a gentle homogenization method like GentleMACS Dissociator combined to QIAzol reagent.

## Methods

### Clinical cohort

Human tissues were collected in the frame of COMET clinical trial, initiated in 2015. The protocol was performed in compliance with various French regulations, such as the provisions relating to biomedical research, the Public Health Act no. 2004-806, the Data Protection Act and the Bioethics Act. It was also performed in accordance with the Declaration of Helsinki and Good Clinical Practice, after approval by the French National Agency for the Safety of Medicines and Health Products (ANSM) and the Sud Méditerranée I Ethics Committee (registration number NCT02861781 on http://www.clinicaltrials.gov). The study was sponsored by the Montpellier University Hospital in collaboration with University of Montpellier. When included into the clinical trial, patients signed a written informed consent for the collection and storage of tissue samples and associated data.

The protocol has included 270 obese patients (grade 2 or 3, body mass index (BMI) ≥ 35 kg/m^2^) from 18 to 65 years old undergoing bariatric surgery (mainly sleeve gastrectomy) and stratified in 3 groups according to their metabolic status: normal insulin sensitivity (Homeostatic Model Assessment of Insulin Resistance [HOMA-IR] < 3), insulin resistance without T2D (HOMA-IR ≥ 3), or T2D (according to American Diabetes Association (ADA) criteria^12^). Insulin-sensitive tissues (SCAT, VAT, liver, skeletal muscle, and epiploic artery tissues) and blood sample derivates (DNA, RNA, plasma and serum) were collected in order to constitute the tissue biobank.

For this study, we have selected 11 female patients, with a mean (± standard deviation) age of 50 ± 10 and BMI of 44.8 ± 5.6, with or without T2D and Non-Alcoholic Steato-Hepatitis (NASH) (Supplementary Table 2). As individual biopsies did not provide sufficient samples, 3 patients out of 11 were selected for each experimental condition, including one diabetic subject.

### Associated data management

Patients’ clinical and biological data were pseudonymized and reported in an electronic Case Report Form (eCRF, ENNOV-CLINICAL), managed by Montpellier University Hospital.

Samples were stored at the Tissue Bank from the Biological Resource Centre (CRB) (BB-0033-00031) and related data were registered on Tumorotek software (from French Ministry of health), designed for biospecimens management. Traceability data related to biopsy sampling, transport and storage were resumed in a written “patient life-sheet”.

### Tissue sampling

All tissue samples were handled following a Standard Operation Procedure (SOP) defined at the beginning of the study. During the surgery, large biopsies of sub-cutaneous and visceral adipose tissues, hepatic and skeletal muscular tissues and epiploic artery (500 mg to 1 g) were collected, immediately sampled on ice into small pieces of 20 (for muscle and liver) or 50 mg (for adipose tissues) and snap-frozen in liquid nitrogen in labelled cryovials within 15 min (for muscle and liver) or 20 min (for adipose tissues). Frozen samples were transferred in a liquid nitrogen dry shipper, the inner wall of which is covered by a porous material that absorbs a certain volume of liquid nitrogen. The container saturates with cold nitrogen gas which allows the maintenance of a stable temperature at − 140 °C, constantly registered by a sensor located in the top of the container. Samples were transported from the surgical block to the Tissue Bank for a long-term storage at − 80 °C, according to the technical protocol (DT-COMET-01), and two procedures (MO-COMET-03, dealing with the collection and preparation of the tissue samples and MO-COMET-06, which concerns the storage of samples at the Tissue Bank).

For evaluation, some samples were alternatively pre-treated in the RNA stabilization solution RNAlater (Sigma-Aldrich) before freezing.

### Tissue disruption

For each condition tested, 50 mg of sub-cutaneous (SCAT) and visceral (VAT) adipose tissues and 20 mg of liver and muscle were used, for a total of 39 samples. The tissues were disrupted using three alternative technologies in combination with two different homogenizing reagents. A summary of the experimental protocol applied to each tissue and patient is shown in Table 2:

1. For bead beating-based homogenization (FastPrep-24 Instrument, MP Biomedicals), samples were put in a 2 mL RNase DNase free tube containing 0.5 mL of 0.5 mm silica/zirconium beads (BioSpec—Cat No. 11079105z) and (i) 300 µL of RLT buffer + β-mercaptoethanol (1%) or (ii) 1 mL of QIAzol buffer. Homogenization was performed on FastPrep-24 using 2–4 cycles at 6 m/s for 30 to 40 s with 5 min incubation time on ice between cycles.
2. For rotor/stator-based homogenization (GentleMACS Dissociator, Miltenyi Biotec), samples were introduced in GentleMACS M tube (Miltenyi Biotec) containing (i) 300µL of RLT buffer + β-mercaptoethanol (1%) or (ii) 1 mL of QIAzol buffer. The tissue was homogenized using the dissociation template RNA 02.01 program.
3. For needle dissociation, trimmed samples were introduced in a 2 mL RNase DNase free tube containing (i) 300µL of RLT buffer + β-mercaptoethanol (1%) or (ii) 1 mL of QIAzol buffer. Homogenization was performed by several back and forth movements through a syringe and needle (18G). Skeletal muscle could not be prepared using this method as it is too compact.
  After homogenization, the samples were centrifuged for 15 min at 3500 rpm at 4 °C in order to separate the tissue lysate from cellular debris.

**Table 2:** Experimental protocols. Sample origin, homogenization buffers (RLT versus QIAzol) and methods (FastPrep for FastPrep Instrument, GentleMACS for GentleMACS Dissociator, Needle for syringe/needle) used were reported for each tissue type.

Tissue	Buffer	Patients	Disruption
VAT	QIAzol	1, 2, 3	Fast-Prep
			GentleMACS
		7, 8, 9	Needle
SCAT		1, 2, 3	Fast-Prep
			GentleMACS
		5, 6, 7	Needle
Liver	RLT	1, 3, 4	Fast-Prep
		7, 10, 11	Needle
		1, 3, 4	GentleMACS
	QIAzol		
Muscle	RLT	1, 3, 4	Fast-Prep
			GentleMACS
	QIAzol		

### RNA extraction

RNA extractions were performed using RNeasy mini kit (Qiagen) for hepatic tissue, RNeasy lipid tissue mini kit (Qiagen) for adipose tissues, and RNeasy fibrous tissue mini kit (Qiagen) for muscular tissue, according to the manufacturer’ instructions. At the end of the extraction protocol, RNA was eluted in 30 µL buffer. Nucleic acid concentration, A260/280 and A260/230 ratios were measured using NanoDrop One/OneC Spectrophotometer (ThermoFischer Scientific).

### RNA integrity assessment

RNA quality was determined by the RNA Integrity Number (RIN), measured by 2100 Bioanalyzer (Agilent Technologies) using RNA 6000 Nano kit, following the manufacturer’s protocol. This automated system is based on electrophoretic separation of RNA, which allows calculating RIN by applying an algorithm on the ratio of 18S/28S ribosomal RNA traces^23^. The RIN number was calculated for the 39 samples subjected to different disruption/homogenization methods. For each tissue type, we used the mean value obtained for samples issued from 3 selected patients (see Table 2).

### Quantitative RT-PCR analysis

RT-qPCR was used to quantify mRNA from three housekeeping genes, such as TATA-box binding protein (TBP, mRNA = 1710 nt; protein = 339 amino acids), β-actin (mRNA = 1761 nt; protein = 375 amino acids) or glyceraldehyde-3 phosphate dehydrogenase (GAPDH mRNA = 1272 nt; protein = 335 amino-acids). For this, 200 ng RNA were reverse transcribed into cDNA in the presence of 1 µg oligo(dT) primers and SuperScript II RNAse H^-^ Reverse Transcriptase (Invitrogen), in a final volume of 20 µL, according to manufacturer’s instructions. Then, 2 µL of 1:5 diluted cDNA was amplified in a final volume of 10 µL by qPCR using LightCycler 480 SybrGreen I Master kit (Roche). We used primers targeting non coding 5′ or 3′UTR regions common in all splice variants described in different tissues (Supplementary Table 3). The following conditions were applied: (i) 95 °C, 5 min, (ii) 40 cycles of 95 °C, 10 s; 60 °C, 15 s; 72 °C, 15 s, and (iii) 95 °C, 10 s on MasterCycler RealPlex (Eppendorf). Technical triplicates were used for qPCR (i.e. independent pipetting of cDNA) to ensure reproducibility of the assay. For each tissue sample and primer pair, the threshold cycle (Ct) was determined and data represented the mean of 3 patients’ samples.

### Statistical analysis

All data are expressed mean ± SEM, unless otherwise stated. Statistical analyses were performed using GraphPad Prism v5.0 (San Diego, CA, USA) or SAS v9.4 (Cary, NC, USA). We analysed the effect of tissue, disruption method and the interaction disruption method × tissue type (adipose or non-adipose tissue) by ANOVA followed by Tukey’s post-hoc tests. For RT-qPCR results, the effect of genes (ACT, GAPDH or TBP) and the interactions gene × tissue type, gene × disruption method and gene × tissue type × disruption method were added to the model. To check the robustness of results, the effect of disruption method was assessed after exclusion of muscle samples (not submitted to the needle approach). The effect of buffer was assessed in non-adipose tissues only, with or without exclusion of the FastPrep method. The effect of tissue origin across the GentleMACS and QIAzol condition was also analysed. Lastly, mixed models with a random intercept by patient were applied to control for the non-independency of observations (analyses were limited to patients 1, 2, 3 or patients 1, 3, 4). Tests were considered statistically significant at P < 0.05 (*P < 0.05; **P < 0.01 and ***P < 0.001).

## Supplementary Information

Supplementary Table 1.

Supplementary Table 2.

Supplementary Table 3.
