# Supplementary material for: Optimization of RNA extraction methods from human metabolic tissue samples of the COMET biobank
Source: Sci Rep. 2021 Oct 25;11:20975. doi: 10.1038/s41598-021-00355-x (PMC8545963; doi:10.1038/s41598-021-00355-x)
Supplement: Supplementary file 1 — Supplementary Table 1. [file 41598_2021_355_MOESM1_ESM.docx]

**Optimization of RNA extraction methods from human metabolic tissue samples of the COMET biobank**

Agathe Nouvel^1^, Jonas Laget^1^, Flore Duranton^1,2^, Jérémy Leroy^1^, Caroline Desmetz^1^, Marie-Dominique Servais^3^, Nathalie de Préville^3^, Florence Galtier^1,4,5^, David Nocca^6^, Nicolas Builles^7^, Sandra Rebuffat^1*^, Anne-Dominique Lajoix^1*^

**Supplemental Table 1.** Estimated least-square means from ANOVA on RT-qPCR results for the three housekeeping genes (full table). The letters indicate significant changes in means (numbers with the same letters are not different). P-value <0.05 are shown in bold. ACT for β-actin, FastPrep for FastPrep Instrument, GentleMACS for GentleMACS Dissociator, Needle for syringe/needle.

| **Dependant variable** | **Ct** | | **Ct/RIN** | |
| --- | --- | --- | --- | --- |
| **Effects and modalities** | **Estimated means** | **P-value** | **Estimated means** | **P-value** |
| **Gene** |  | **<0.0001** |  | **<0.0001** |
| ACT | 18.4^c^ |  | 2.7^b^ |  |
| GAPDH | 29.4^a^ |  | 4.4^a^ |  |
| TBP | 28.5^b^ |  | 4.2^a^ |  |
| **Tissue,** |  | 0.84 |  | **0.04** |
| Liver | 25.9^a^ |  | 4.5^a^ |  |
| Muscle | 26.1^a^ |  | 3.8^b^ |  |
| SCAT | 25.0^ab^ |  | 3.3^b^ |  |
| VAT | 24.8^b^ |  | 3.3^b^ |  |
| **Disruption,** |  | **0.005** |  | **0.003** |
| FastPrep | 24.9^b^ |  | 3.9^a^ |  |
| GentleMACS | 25.1^b^ |  | 3.3^b^ |  |
| Needle | 26.3^a^ |  | 3.9^a^ |  |
| **Tissue Type x Disruption** |  | 0.55 |  | 0.08 |
| Adipose tissue, FastPrep | 24.5^b^ |  | 3.9^ab^ |  |
| Adipose tissue, GentleMACS | 24.4^b^ |  | 2.9^c^ |  |
| Adipose tissue, Needle | 25.8^ab^ |  | 3.3^bc^ |  |
| Non-adipose tissue, FastPrep | 25.3^ab^ |  | 4.0^ab^ |  |
| Non-adipose tissue, GentleMACS | 25.9^a^ |  | 3.7^ab^ |  |
| Non-adipose tissue, Needle | 26.8^a^ |  | 4.6^a^ |  |
| **Gene x Tissue Type,** |  | **0.0002** |  | 0.45 |
| ACT, adipose tissue | 17.1^d^ |  | 2.3^c^ |  |
| ACT, non-adipose tissue | 19.8^c^ |  | 3.2^b^ |  |
| GAPDH, adipose tissue | 29.7^a^ |  | 4.1^ab^ |  |
| GAPDH, non-adipose tissue | 29.2^ab^ |  | 4.7^a^ |  |
| TBP, adipose tissue | 27.9^b^ |  | 3.8^ab^ |  |
| TBP, non-adipose tissue | 29.0^ab^ |  | 4.7^a^ |  |
| **Gene x Disruption** |  | 0.06 |  | 0.98 |
| ACT, FastPrep | 18.5^c^ |  | 3.0^bc^ |  |
| ACT, GentleMACS | 18.5^c^ |  | 2.4^c^ |  |
| ACT, Needle | 18.2^c^ |  | 2.8^bc^ |  |
| GAPDH, FastPrep | 28.5^ab^ |  | 4.6^a^ |  |
| GAPDH, GentleMACS | 28.9^ab^ |  | 3.8^ab^ |  |
| GAPDH, Needle | 30.3^a^ |  | 4.7^a^ |  |
| TBP, FastPrep | 27.7^b^ |  | 4.5^a^ |  |
| TBP, GentleMACS | 28.1^ab^ |  | 3.7^ab^ |  |
| TBP, Needle | 29.6^ab^ |  | 4.5^a^ |  |
| **Tissue Type x Gene x Disruption** |  | 0.28 |  | 0.99 |
| Adipose tissue, ACT, FastPrep | 16.8^d^ |  | 2.7^abc^ |  |
| Adipose tissue, ACT, GentleMACS | 17.3^cd^ |  | 2.1^c^ |  |
| Adipose tissue, ACT, Needle | 17.2^cd^ |  | 2.3^bc^ |  |
| Adipose tissue, GAPDH, FastPrep | 29.1^ab^ |  | 4.7^a^ |  |
| Adipose tissue, GAPDH, GentleMACS | 28.7^ab^ |  | 3.4^abc^ |  |
| Adipose tissue, GAPDH, Needle | 31.3^a^ |  | 4.2^a^ |  |
| Adipose tissue, TBP, FastPrep | 27.8^b^ |  | 4.5^a^ |  |
| Adipose tissue, TBP, GentleMACS | 27.3^b^ |  | 3.3^abc^ |  |
| Adipose tissue, TBP, Needle | 28.7^ab^ |  | 3.8^ab^ |  |
| Non-adipose tissue, ACT, FastPrep | 20.3^c^ |  | 3.3^abc^ |  |
| Non-adipose tissue, ACT, GentleMACS | 19.7^c^ |  | 2.9^abc^ |  |
| Non-adipose tissue, ACT, Needle | 19.3^cd^ |  | 3.4^abc^ |  |
| Non-adipose tissue, GAPDH, FastPrep | 27.9^b^ |  | 4.5^a^ |  |
| Non-adipose tissue, GAPDH, GentleMACS | 29.0^ab^ |  | 4.2^a^ |  |
| Non-adipose tissue, GAPDH, Needle | 30.5^ab^ |  | 5.4^a^ |  |
| Non-adipose tissue, TBP, FastPrep | 27.5^b^ |  | 4.4^a^ |  |
| Non-adipose tissue, TBP, GentleMACS | 28.9^ab^ |  | 4.2^a^ |  |
| Non-adipose tissue, TBP, Needle | 30.5^ab^ |  | 5.4^a^ |  |
