# Supplementary material for: Optimization of RNA extraction methods from human metabolic tissue samples of the COMET biobank
Source: Sci Rep. 2021 Oct 25;11:20975. doi: 10.1038/s41598-021-00355-x (PMC8545963; doi:10.1038/s41598-021-00355-x)
Supplement: Supplementary file 2 — Supplementary Table 2. [file 41598_2021_355_MOESM2_ESM.docx]

**Optimization of RNA extraction methods from human metabolic tissue samples of the COMET biobank**

Agathe Nouvel^1^, Jonas Laget^1^, Flore Duranton^1,2^, Jérémy Leroy^1^, Caroline Desmetz^1^, Marie-Dominique Servais^3^, Nathalie de Préville^3^, Florence Galtier^1,4,5^, David Nocca^6^, Nicolas Builles^7^, Sandra Rebuffat^1*^, Anne-Dominique Lajoix^1*^

**Supplemental table 2. Patient’s characteristics.** Patients were pseudonymized from 1 to 11. BMI: body mass index; T2D: type 2 diabetes; NASH: non-alcoholic steato-hepatitis.

| Patient ID | Age at surgery | BMI (kg/m^2^) | T2D | NASH |
| --- | --- | --- | --- | --- |
| 1 | 46 | 45.12 | no | no |
| 2 | 26 | 38.3 | no | no |
| 3 | 56 | 45.12 | yes | no |
| 4 | 62 | 41.53 | no | no |
| 5 | 43 | 43.71 | no | no |
| 6 | 59 | 44.38 | no | no |
| 7 | 54 | 40.04 | yes | yes |
| 8 | 44 | 52.03 | no | no |
| 9 | 59 | 47.03 | no | yes |
| 10 | 51 | 38.75 | no | no |
| 11 | 46 | 56.69 | no | no |
