# Supplementary material for: Optimization of RNA extraction methods from human metabolic tissue samples of the COMET biobank
Source: Sci Rep. 2021 Oct 25;11:20975. doi: 10.1038/s41598-021-00355-x (PMC8545963; doi:10.1038/s41598-021-00355-x)
Supplement: Supplementary file 3 — Supplementary Table 3. [file 41598_2021_355_MOESM3_ESM.docx]

**Optimization of RNA extraction methods from human metabolic tissue samples of the COMET biobank**

Agathe Nouvel^1^, Jonas Laget^1^, Flore Duranton^1,2^, Jérémy Leroy^1^, Caroline Desmetz^1^, Marie-Dominique Servais^3^, Nathalie de Préville^3^, Florence Galtier^1,4,5^, David Nocca^6^, Nicolas Builles^7^, Sandra Rebuffat^1*^, Anne-Dominique Lajoix^1*^

**Supplemental table 3.** Primers used for quantitative RT-PCR. The sequence of each primer used, the length of the amplicon and its localization in mRNA was indicated. TBP: TATA-box binding protein, GAPDH: glyceraldehyde-3 phosphate deshydrogenase.

| **Genes ID** | **Primers** | **Sequence** | **Amplicon (bp)** | **Localisation** |
| --- | --- | --- | --- | --- |
| **TBP** | forward | CATTATCAACGCGCGCCAG | 123 | Exon 1 and 2  5’UTR |
|  | reverse | ACCCTGGGTCACTGCAAAG |  |  |
| **β-ACTIN** | forward | GTCATTCCAAATATGAGATGCGT | 121 | Exon 6  3’UTR |
|  | reverse | GCTATCACCTCCCCTGTGTG |  |  |
| **GAPDH** | forward | GAAATGAATGGGCAGCCGTT | 133 | Exon 1  5’UTR |
|  | reverse | ATCACCCGGAGGAGAAATCG |  |  |
